# Theta burst stimulation of the visual cortex does not alter contrast sensitivity under optically induced blur

**DOI:** 10.1371/journal.pone.0341942

**Published:** 2026-09-01

**Authors:** Crystal Suei Cheng Wong, Ken Wei Sheng Tan, Tingni Li, Linden Gaultney, Benjamin Thompson, Tsz Wing Leung, Adela So Yun Park

**Affiliations:** 1 School of Optometry, The Hong Kong Polytechnic University, Hong Kong SAR, China; 2 Centre for Eye & Vision Research, Hong Kong Science Park, Hong Kong SAR, China; 3 School of Optometry and Vision Science, University of Waterloo, Canada; Hangzhou Normal University, CHINA

## Abstract

This study investigated whether a single session of repetitive transcranial magnetic stimulation (TMS), specifically theta burst stimulation (TBS), acutely modulates visual function under conditions of induced optical defocus. Twelve adults with normal or corrected-to-normal vision participated in a pre-registered, within-subject, double-blind, sham-controlled study. Participants first adapted to an experimentally induced + 1.50D optical blur for 10 minutes. Following this, stimulation was applied to the primary visual cortex according to a pre-randomised allocation. Stimulation consisted of continuous TBS (cTBS), intermittent TBS (iTBS), or sham stimulation, with each condition applied to all participants over separate visits. Visual acuity (VA) and contrast discrimination thresholds were measured under the induced blur condition immediately before and after stimulation. Repeated measures ANOVA revealed no significant interaction between stimulation condition and time for either contrast discrimination threshold (F(2,22) = 0.03, *p* = 0.96, partial ηp² = 0.003) or VA (F(2,22) = 0.12, *p* = 0.88, partial ηp² = 0.011). Although a significant main effect of time was observed for VA (F(1,11) = 6.30, *p* = 0.029, partial ηp² = 0.364), reflecting an improvement across all conditions, there were no differential effects attributable to active cTBS or iTBS compared to sham. Consequently, a single session of TBS applied to the visual cortex did not result in a measurable modulation of visual performance under acute optical defocus in adults with typical vision. Rapid homeostatic mechanisms activated during short-term blur adaptation may reduce the susceptibility of the visual cortex to the acute effects of TBS.

## Introduction

Myopia (near-sightedness) is a leading cause of distance vision impairment globally, with prevalence projected to affect nearly half of the global population by 2050 [1]. Myopia selectively attenuates high-frequency spatial information, thereby reducing the signal available for cortical processing [2]. Visual performance is fundamentally dependent on both the quality of the retinal input and the efficiency of subsequent visual neural processing. Deficits arising from optical blur may, in principle, be partly compensated for by enhancing neural processing downstream of the optics. The capacity of the adult visual system to reorganise, a mechanism known as neuroplasticity, forms the fundamental basis for such functional improvements [3].

Perceptual learning (PL) strongly supports the notion that visual function can be neurally enhanced in specific visual tasks trainings [4]. Training-based improvements in contrast sensitivity (CS) and visual acuity (VA) have been repeatedly demonstrated in conditions involving degraded retinal image quality, including myopia [5,6] and presbyopia [7]. While PL achieves these functional improvements predominantly through reweighting of sensory information for perceptual decision-making [8,9] rather than fundamentally altering the initial sensory representations, a similar or accelerated enhancement may be achievable through direct modulation of cortical activity.

Non-invasive brain stimulation (NIBS), including both transcranial magnetic stimulation (TMS) and transcranial electrical stimulation (tES), offers a means to modulate cortical excitability and network dynamics, thereby facilitating changes in neuroplasticity [10]. Importantly, when paired with PL, NIBS can further amplify and sustain training effects [11,12]. For example, combining transcranial random noise stimulation (tRNS, a subtype of tES) with PL was shown to yield significant, prolonged improvements in uncorrected VA and CS in young adults with mild myopia [13]. Similarly, our previous work demonstrated that a two-week intervention combining PL with either visual cortex tRNS or TMS resulted in sustained improvements exceeding one logMAR line in binocular distance-corrected near VA in older adults with presbyopia [14]. These studies highlight the potential of NIBS to accelerate, enhance, and prolong the effects of PL, demonstrating its capacity to enhance plasticity in the adult visual cortex.

The acute effects of NIBS alone on visual function have also been investigated. These studies have employed various NIBS protocols, including theta burst stimulation (TBS), a patterned form of repetitive TMS (rTMS), that can rapidly induce robust and lasting changes in cortical excitability [15]. In the motor cortex, continuous TBS (cTBS) is commonly observed to induce inhibitory effects, whereas intermittent TBS (iTBS) typically induces facilitatory effects [15]. However, this inhibitory-facilitatory dichotomy is not clear-cut, with evidence showing individual and contextual variability [16].

The effects of TBS on the visual cortex are not uniform. Physiologically, cTBS applied to V1 elevates phosphene thresholds (PT, a perceptual index of visual cortical excitability), consistent with reduced excitability [17,18], while iTBS has little to no effect on PT [17,19]. Behaviourally, the outcomes are even more complicated. cTBS over V1 has been shown to decrease CS in some instances [18], yet improves it under certain spatial configurations in others [20]. These inconsistencies suggest that visual cortex TBS outcomes may be more nuanced than the simpler inhibitory-excitatory dichotomy observed in the motor system and could depend heavily on pre-stimulation brain states or specific task demands.

Despite these inconsistent results in healthy populations, cTBS protocols have demonstrated relatively consistent therapeutic efficacy in visually impaired clinical populations. For example, studies applying cTBS over V1 have shown reliable improvements in CS [21] and VA [22,23] for adults with amblyopia. The longevity of these improvements is substantial, with retention reported up to 78 days following cTBS [21] and averaging 181 days post-TBS in recent work [23]. The enhanced effects of NIBS in individuals whose visual cortices have undergone chronic, long-term maladaptive neuroplasticity aligns with the theory of homeostatic plasticity. This theory posits that the cortex regulates its average activity and may therefore be more amenable to plasticity induction when operating outside its optimal equilibrium [24].

Given this context, the present study investigated whether an acute perturbation of sensory input, with experimentally induced blur, is sufficient to activate the homeostatic mechanisms required for NIBS-driven functional improvement. This is an important knowledge gap because prior standalone TBS studies in typically sighted individuals have almost exclusively been conducted under conditions of optimal vision, leaving the effect of acute degradation unknown.

The present study employed experimentally induced optical defocus in participants with normal or corrected-to-normal vision to induce an acute vision deficit across participants. This allowed us to isolate the immediate capacity of the adult visual cortex for NIBS-driven plasticity, independent of PL effects. A within-subject design was employed, with contrast discrimination threshold (hereafter referred to as contrast threshold) and VA as outcome measures, to directly compare the effects of cTBS and iTBS protocols. We hypothesised that active TBS, but not sham stimulation, would modulate visual function. Specifically, despite the acknowledged nuanced effects of TBS, we hypothesised that cTBS would decrease VA and contrast threshold, whereas iTBS would improve both measures based on established TBS literature. The work is part of a longer-term research program that will inform the design of future clinical trials utilising NIBS as a rapid, standalone intervention for the loss of visual sensitivity caused by refractive error.

## Methods

### Study design and participants

#### Study design.

The study employed a within-subject, double-blind, sham-controlled, cross-over design spanning three separate visits, each separated by a minimum washout period of 24 hours. The procedures followed a pre-registered protocol (registered on 09/05/2023; OSF registries: https://doi.org/10.17605/OSF.IO/QDMR4) detailing the hypotheses, methodology, and analysis plan. Deviations from the pre-registered protocol included the exclusion of sweep VEP data; despite adherence to ISCEV standards using full-field vertical square wave gratings, the induced blur and 60% contrast conditions resulted in excessive noise and low VEP amplitudes, making the data unusable for analysis. Furthermore, while the contrast discrimination threshold was the primary pre-registered outcome, the VA analysis was adopted post-hoc. VA was originally collected as a blur adaptation check, and the final stabilised value (the last time point of the adaptation period, at 10 or 12 minutes) was subsequently used as a dependent variable in the analysis. We confirm that the analysis plan for the primary contrast threshold outcome was not modified after inspecting the data.

The order of the TMS interventions (cTBS, iTBS, sham) was randomised across participants using a computer-generated pseudorandom sequence.

#### Screening, eligibility, and consent.

Prior to the main experiment, participants underwent a vision screening to confirm study eligibility. This involved assessments of best-corrected distance visual acuity (BCDVA), objective and subjective refraction, and stereopsis. A TMS safety questionnaire [25] was used to screen for ocular and systemic diseases and contraindications to TMS. Eligibility was based on the following inclusion/exclusion criteria:

Inclusion criteria:

- Aged 18–35 years (inclusive)
- BCDVA of 0.10 logMAR or better in both eyes
- Refractive error within ± 4.00 dioptre (D) spherical equivalent
- Normal stereoacuity (< 70” by Randot circles of The Randot test [Stereo Optical Co., Chicago, IL]).

Exclusion criteria:

- Self-reported ocular pathologies impairing vision
- Any neurological conditions affecting vision or visual functions
- Inability to comprehend the psychophysics tasks of this study
- Any contraindications to NIBS as assessed by a safety questionnaire

Participants were recruited between 10 May 2023 and 13 December 2023. All participants provided written informed consent prior to participation. The study protocol was approved by The Hong Kong Polytechnic University Institutional Review Board (HSEARS20210811006) and adhered to the tenets of the Declaration of Helsinki. Participants received remuneration (HKD $200/session) for their time.

### Experimental procedure

**Fig 1:**
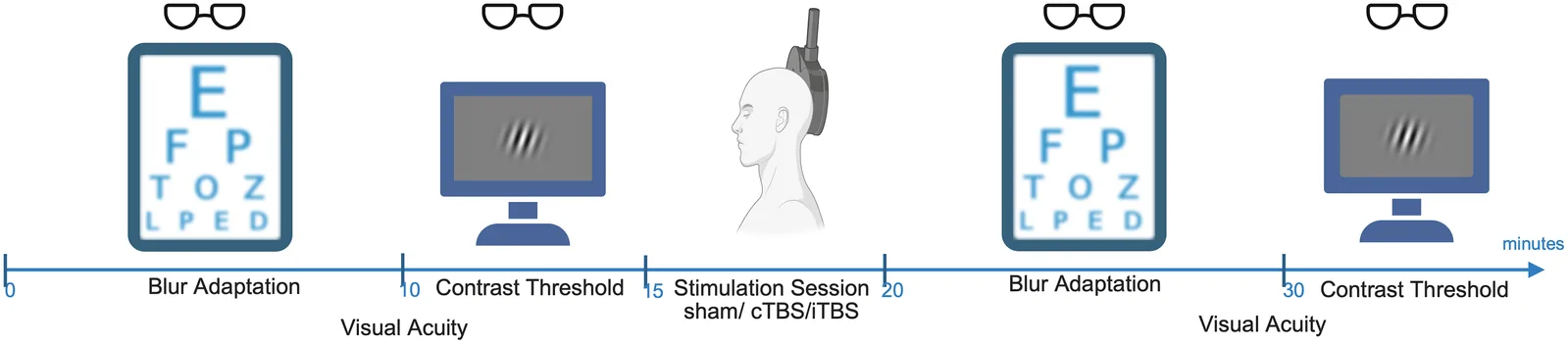
A schematic flowchart illustrating the design of the study.

Fig 1 illustrates the experimental workflow. Each session followed the same structure consisting of pre-stimulation assessment, TBS intervention, and post-stimulation assessment.

Pre-stimulation assessment: Participants began by wearing trial lenses to induce the + 1.50D optical defocus (calculated based on their subjective refractive correction and viewing distance). They then underwent a 10-minute blur adaptation period, based on previous findings that adaptation stabilises within this timeframe [26]. Following this adaptation, the stabilised VA and baseline contrast thresholds were measured as the “pre-stimulation” values.

- Intervention: Over the course of the study, participants received all three TMS protocols (cTBS, iTBS or sham [cTBS/iTBS]). Only one protocol was administered per visit based on their predetermined random allocation. Stimulation was administered in a well-lit room, and no induced blur was present during TMS administration as participants did not wear their trial lenses and kept their eyes closed throughout stimulation.
- Post-stimulation assessment: Upon TMS cessation, the post-stimulation assessment would begin. Participants re-wore the trial lenses inducing the + 1.50D defocus and repeated the 10-minute blur adaptation protocol to ensure a stable state of adaptation. After adaptation, stabilised VA and contrast thresholds were measured as the post-stimulation values. All post-stimulation assessments were completed within 30 minutes of TMS cessation.

Each participant underwent three TMS sessions on separate days. During each session, participants first experienced a 10-minute blur adaptation period, during which they wore trial lenses that induced a + 1.50D optical defocus. During these 10-minutes, VA was measured at the 5-, 7- and 10-minute mark to ensure adequate blur adaptation. Adaptation was considered complete when VA improved by less than one line between the 7- and 10-minute measurements; otherwise, it was remeasured at 12 minutes. Following this, a baseline measurement of contrast threshold was obtained. Upon completion of baseline measurements, administration of stimulation with either iTBS, cTBS or sham would begin, participant removed the trial lenses and kept their eyes closed during stimulation. After stimulation, participants then wore the same trial lenses inducing the + 1.50D defocus and repeated the 10-minute blur adaptation protocol to ensure a stable state of adaptation. Post-stimulation VA and contrast thresholds were measured, with all assessments completed within 30 minutes of TMS.

#### Participants.

A total of sixteen individuals were screened for eligibility. Of these, thirteen participants (8 females, 5 males; mean age ± SEM: 28.6 ± 1.2 years [range: 23–35 years]) met all inclusion criteria, consented to participate, and completed the study protocol. Data from one participant (female) were subsequently excluded from the final analysis due to a technical error involving incorrect blinding codes. The final sample used for data analysis consisted of twelve participants (7 females, 5 males; mean age ± SEM: 28.1 ± 1.0 years [range: 23–33 years]). The clinical, demographic, and PT characteristics of the final analysed sample are detailed in Table 1.

**Table 1:** Clinical, demographic, and cortical excitability characteristics of the final analysed subjects (n = 12).

ID	Age (years)	Sex	Refraction (D)	BCVA (logMAR)	Stereopsis (arcsec)	Phosphene Threshold (% MSO)
01	31	Male	RE: + 1.00 / −0.75 × 170 LE: + 0.75 / −0.50 × 170	RE: −0.1 LE: −0.1	40	42%
02	23	Male	RE: + 1.00 / −0.25 × 95 LE: + 1.00 DS	RE: −0.08 LE: −0.1	30	35%
03	29	Female	RE: −2.50 / −0.25 × 180 LE: −1.00 / −0.75 × 15	RE: −0.08 LE: −0.08	20	40%
04	31	Female	RE: −0.75 / −1.5 × 20 LE: −0.25 / −1.25 × 154	RE: −0.1 LE: −0.1	30	43%
05	27	Female	RE: + 1.25 / −0.75 × 75 LE: + 1.00 / −0.75 × 105	RE: −0.1 LE: −0.1	20	26%
06	25	Female	RE: −1.50 DS LE: −1.75 DS	RE: −0.1 LE: −0.1	50	41%
07	32	Male	RE: planoLE: plano	RE: −0.1 LE: −0.1	20	42%
08	31	Female	RE: plano / −0.50 × 90LE: + 0.25 DS	RE: −0.1 LE: −0.1	25	64%
09	23	Female	RE: −1.75 / −0.25 × 5LE: −0.50 / −0.50 × 10	RE: −0.1 LE: −0.08	50	52%
10	27	Male	RE: −3.25 / −1.5 × 175 LE: −3.25 / −1.25 × 10	RE: −0.1 LE: −0.1	50	16%
11	26	Female	RE: −1.50 / −0.5 × 180 LE: −1.00 / −1.00 × 180	RE: −0.08 LE: −0.06	25	50%
12	33	Male	RE: −3.00 / −0.5 × 5 LE: −2.00 / −0.25 × 180	RE: −0.1 LE: −0.1	20	34%

Abbreviations: D = Dioptre, RE = Right Eye, LE = Left Eye, logMAR = logarithm of the Minimum Angle of Resolution, MSO = Maximum Stimulator Output.

### Transcranial magnetic stimulation setup and hotspot localisation

TMS was administered using a MagPro X100 stimulator (MagVenture, Farum, Denmark) connected to a figure-eight coil (MagVenture, Farum, Denmark; model C-B70 for single pulse PT determination; Cool-B70 A/P for TBS protocols). Coil positioning was guided by a frameless stereotaxic neuronavigation system (Localite, Bonn, Germany). The system co-registered the individually identified V1 hotspot location from the initial visit to a standard, anatomical head model provided by the software, ensuring consistent targeting across all sessions.

### Hotspot localisation and phosphene threshold determination

The primary visual cortex (V1) stimulation site, referred to as the ‘hotspot’, was determined individually. Starting 2 cm dorsal to the inion, single TMS pulses were delivered systematically over a 5 x 5 cm grid, with the lower edge of the grid centred on the inion and the coil handle oriented upward and parallel to the subject’s spinal cord [21]. The initial stimulation intensity was set to 60% of the maximum stimulator output (MSO). Intensity was increased in 2% increments until a phosphene was reliably perceived or a ceiling of 70% MSO was reached. The hotspot was defined as the scalp location where the participant reported the most vivid and consistent phosphene and was marked using the neuro-navigation system [27].

Following hotspot identification, the individual’s PT was determined. PT was defined as the lowest TMS intensity required to reliably elicit phosphenes in 5 out of 10 consecutive single pulses delivered to the hotspot, determined by systematically varying the stimulation intensity [28,29].

#### Theta burst stimulation (TBS) protocols.

TBS was administered in three conditions: iTBS, cTBS, and sham. Stimulation was delivered using a Cool-B70 A/P coil, which incorporates both an active and a placebo side that are physically indistinguishable to the experimenter, thereby ensuring experimenter blinding. Stimulation intensity was set at 80% of the individual’s PT or 45% of the MSO, whichever was lower. The parameters were defined as follows:

- *iTBS:* A 2-second train of TBS was repeated every 10 seconds for a total duration of 190 seconds, yielding 600 pulses.
- *cTBS:* A 40-second continuous train of TBS, also yielding 600 pulses.
- *Sham:* Delivered using the same coil, with the coil flipped so that the placebo side contacted the scalp, resulting in no effective magnetic field reaching the cortex.

### Blinding and randomisation

A double-blinded design was maintained using a pre-generated randomization list, with associated blinding codes, managed by a team member naïve to the experimental aims. These codes specified coil orientation (active vs. placebo side), ensuring that the operator remained blinded to stimulation condition. This list randomised the order of stimulation conditions (iTBS, cTBS, and sham) across the three visits. To preserve operator blinding, the sham condition was randomly assigned to be either “sham-iTBS” or “sham-cTBS”. This ensured that while operators could distinguish between the rhythmic patterns of iTBS and cTBS, they remained blinded to whether the stimulation delivered was active or sham.

### Induced optical blur and visual acuity

To induce a + 1.50D optical blur, participants wore trial lenses over their subjective refraction in a pair of trial frames. Participants then underwent a 10-minute blur adaptation period prior to pre- and post-stimulation measurements. During this period, they viewed a logMAR ETDRS vision chart (Good-Lite®, model: ESV3000 ETDRS Illuminated Cabinet) at 4 meters. To confirm stabilisation and minimise the potential confounding effect of visual adaptation, VA was monitored at 5-, 7-, and 10-minute time points (during the 10-minute blur adaptation period). Adaptation was considered complete when VA improved by less than one line between the 7- and 10-minute measurements; otherwise, it was remeasured at 12 minutes. The final stabilised VA measurement (taken at 10 or 12 minutes) was analysed as a dependent variable alongside contrast threshold.

### Contrast discrimination threshold measurement

#### Psychophysical setup and stimuli.

Participants viewed visual stimuli binocularly in a darkened room (0.058 cd/m^2^). Stimuli were generated in Python and presented in PsychoPy (v2021.2.3) [30,31] on an Intel® Core i7-11700K, 3.6 GHz, 8-Core CPU (Intel Corporation, Santa Clara, CA, USA) with 32 GB of RAM and drawn on the frame buffer of an NVIDIA GeForce RTX 3080Ti GPU (NVIDIA, Santa Clara, CA, USA). Visual stimuli were presented on a gamma linearised Cambridge Research Systems Display++ monitor (screen resolution: 1920 × 1080, refresh rate: 120 Hz; Cambridge Research Systems Rochester, UK) at a viewing distance of 1 m. Viewing distance was maintained with a head and chin rest.

Contrast threshold was measured using a two-alternative forced-choice (2AFC) orientation-discrimination task. The central stimulus was a circular Gabor patch (luminance-defined sinusoidal gratings weighted by a Gaussian envelope) with a carrier spatial frequency of 10 cycles per degree (cpd), chosen because this frequency approximates the cut-off frequency after the induced + 1.50D blur [32]. The Gaussian envelope had σ = 0.33°, giving a diameter of the envelope of the Gabor patch a half maximum contrast of 7.84°. The carrier grating was in sine phase relative to the centre of the envelope; this ensured that the midpoint of the Gabor patch was therefore at background luminance (56.12 cd/m^2^). The Gabor patch was always oriented in one of two orientations: either at −45° or +45° (relative to vertical), and participants indicated their perceived orientations using the left or right arrow keys of a keyboard, respectively.

### Procedure and data fitting

Each trial began with a black central fixation cross (0.2°) presented for a randomly jittered duration (500–750 ms), followed by the target Gabor patch being presented for 160 ms (in one of two orientations). A brief auditory tone (150 ms) was played synchronously on stimulus onset to inform participants that the target had been presented. Afterward, a blank screen was shown for 1000 ms for response registration; null responses (i.e., no response within the 1000 ms window) were treated as incorrect. Contrast levels were controlled using the Method of Constant Stimuli. Nine logarithmically spaced contrast levels (range: 0.01–0.6 Michelson contrast) were presented. Each contrast level was presented 20 times (10 times for each orientation), resulting in a total of 180 trials per block, administered in a pseudo-randomised order. Participants were familiarised with the task by including practice trials both with and without induced optical blur, during the initial screening session. The contrast discrimination task was administered before and after each active or sham TMS session.

For data analysis, proportion correct was computed at each contrast level, pooled across orientations. A four-parameter Weibull psychometric function was fitted using weighted nonlinear least squares (SciPy curve_fit), with threshold (κ) and slope (α) as free parameters; the guess rate (γ) was fixed at 0.5, corresponding to chance performance in the two-alternative forced-choice task, and the lapse rate (λ) was fixed at 0.02. Contrast threshold was defined as the stimulus level corresponding to 76% correct performance. This threshold, representing the inverse of contrast sensitivity [33], was the key psychophysical outcome measure used to evaluate any acute changes in visual performance following TBS. Weibull functions fitted the data well (median R² = 0.88).

### Data processing and analysis

To evaluate the effects of TBS on VA and contrast thresholds, a separate two-way repeated measures (RM) ANOVA was conducted for each outcome measure. The within-subject factors were stimulation (cTBS vs. iTBS vs. sham) and time (pre- vs. post-stimulation). Data analysis was performed using SPSS Statistics (version 28.0, IBM Corp., New York, NY, USA). The statistical significance level for all analyses was set at α < 0.05. Partial eta squared (ηp²) is reported for all effects. Normality of the change scores was assessed with the Shapiro-Wilk test and sphericity with Mauchly’s test; Greenhouse-Geisser corrected degrees of freedom are reported where sphericity was violated. To quantify evidence for the null hypothesis, the active versus sham change was additionally evaluated with a Bayesian paired analysis reporting the Bayes factor in favour of the null (BF_01_). A sensitivity analysis indicated that, with twelve participants at α = 0.05 and 80% power, only a large within-subject effect (d_z_ of approximately 0.89) could be detected.

## Results

RM ANOVA on pre-stimulation contrast thresholds showed no statistically significant baseline differences (F(2,22) = 0.36, *p* = 0.69, partial ηp² = 0.032) between the three testing sessions. Likewise, RM ANOVA on pre-stimulation VA revealed no statistically significant baseline differences (F(2,22) = 2.49, *p* = 0.10, partial ηp² = 0.185). These results indicate that baseline measurements were stable.

### Contrast discrimination thresholds

**Fig 2:**
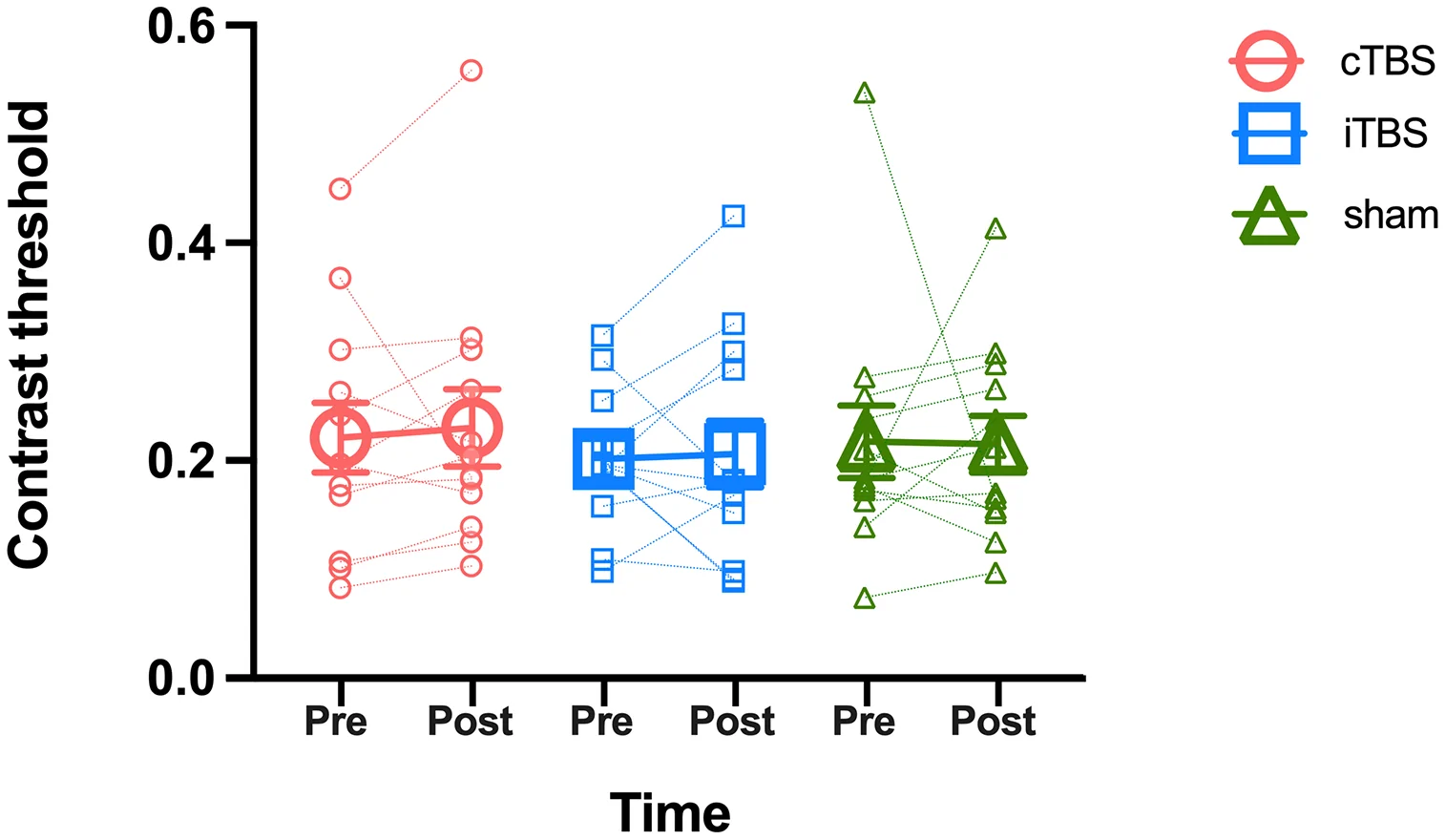
Mean ± SEM contrast thresholds before (Pre) and after (Post) cTBS, iTBS, and sham stimulation. Conditions are indicated by the different coloured symbols and individual participant data are presented by connected dots. Analyses indicated no statistically significant differences in contrast thresholds from pre to post for all three conditions.

A two-way RM ANOVA on contrast thresholds revealed a statistically non-significant interaction of Stimulation × Time (F(2,22) = 0.03, *p* = 0.96, partial ηp² = 0.003), indicating that the differences in pre- and post-TBS measurements were consistent across the three stimulation conditions. No main effects of Stimulation (F(2,22) = 1.07, *p* = 0.35, partial ηp² = 0.089) or Time (F(1,11) = 0.07, *p* = 0.79, partial ηp² = 0.007) were observed. As shown in Fig 2, contrast thresholds remained stable from pre- to post-stimulation across all three conditions. The mean ± SEM contrast thresholds were: cTBS (Pre: 0.22 ± 0.03, 95% CI 0.15–0.29 vs. Post: 0.23 ± 0.03, 95% CI 0.15–0.31), iTBS (Pre: 0.20 ± 0.01, 95% CI 0.16–0.24 vs. Post: 0.20 ± 0.03, 95% CI 0.14–0.27), and sham (Pre: 0.21 ± 0.03, 95% CI 0.15–0.29 vs. Post: 0.21 ± 0.02, 95% CI 0.16–0.27). The pre-to-post change did not differ between the active and sham conditions; the Bayesian analysis provided moderate evidence against a differential effect (BF_01_ = 3.41 for cTBS vs. sham and 3.45 for iTBS vs. sham). Sphericity was not violated (Mauchly, *p* = 0.064) and Greenhouse-Geisser correction did not alter this conclusion. Change scores were normally distributed for the active conditions; the sham change deviated from normality (Shapiro-Wilk, *p* = 0.009), a Wilcoxon signed-rank test confirmed the null result.

### Visual acuity

**Fig 3:**
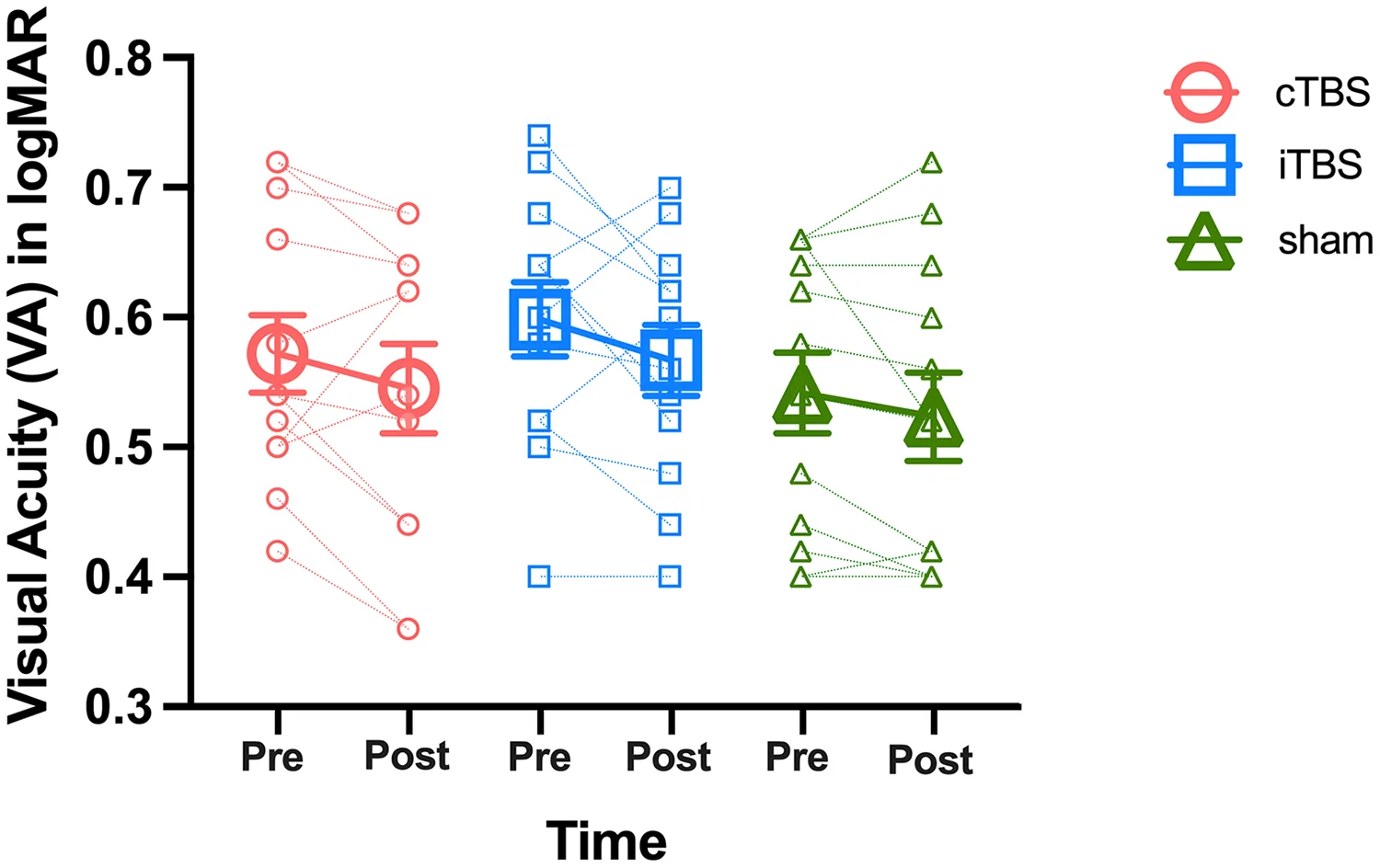
Mean ± SEM logMAR VA before (Pre) and after (Post) cTBS, iTBS, and sham stimulation. Conditions are indicated by the different coloured symbols and individual participant data are presented by connected dots. In logMAR units, a change of 0.1 corresponds to one line on a VA chart. Analyses indicated a statistically significant improvement in VA from pre to post for all three conditions.

Consistent with the contrast threshold results, the analysis of VA yielded no significant interaction of Stimulation × Time (Two-way RM ANOVA, F(2, 22) = 0.12, *p* = 0.88, partial ηp² = 0.011). The main effect of Stimulation was not observed to be statistically significant (F(2,22) = 2.95, *p* = 0.07, partial ηp² = 0.212). However, a significant main effect of Time was observed (F(1,11) = 6.30, *p* = 0.029, partial ηp² = 0.364), indicating a small but consistent improvement in VA from pre- to post-stimulation across all three conditions (mean ± SEM VA pre: 0.57 ± 0.01 and post: 0.54 ± 0.01). As illustrated in Fig 3, VA was similar across all groups. Specifically, VA (logMAR) values were: cTBS (pre: 0.57 ± 0.03, 95% CI 0.51–0.64 vs. post: 0.54 ± 0.03, 95% CI 0.47–0.62), iTBS (pre: 0.59 ± 0.02, 95% CI 0.54–0.66 vs. post: 0.56 ± 0.02, 95% CI 0.51–0.63), and sham (pre: 0.54 ± 0.03, 95% CI 0.47–0.61 vs. post: 0.52 ± 0.03, 95% CI 0.45–0.60). The improvement in VA did not differ between the active and sham conditions; the Bayesian analysis provided moderate evidence against a differential effect (BF_01_ = 3.30 for cTBS vs. sham and 3.08 for iTBS vs. sham). Sphericity was not violated (Mauchly, *p* = 0.59), and all change scores were normally distributed (Shapiro-Wilk, all *p* > 0.15).

## Discussion

This study tested the hypothesis that visual cortex cTBS and iTBS would differentially enhance or inhibit the visual system’s short-term plasticity response to induced retinal image blur. Our findings indicated that there were no changes to contrast threshold for all three stimulation conditions. While a small, statistically significant improvement in VA was observed over time (0.03 logMAR unit, corresponding to 1.5 letters on a standard logMAR chart), this improvement was indistinguishable between active (cTBS or iTBS) or sham stimulation. These results suggest that a single session of TBS was insufficient to alter visual process underlying induced acute blur adaptation.

State dependency suggests that the effects of TMS are not uniform but depend critically on the functional state of the targeted cortical network at the time of stimulation [34,35]. The absence of a measurable differential effect in our study may indicate that the visual cortex had reached a homeostatic equilibrium prior to the intervention [36]. Applying TBS to this “balanced” state may have resulted in an equal modulation of activity across all relevant neural populations, leading to a lack of specific perceptual change. Homeostatic control in V1 is mediated by the inhibitory neurotransmitter GABA; Lunghi and colleagues [36] provided direct evidence that short-term sensory deprivation triggers a reduction in GABA concentration in V1, a compensatory response designed to maintain constant neural activity and induce a perceptual boost. We posit that the 10-minute period of blur adaptation in our protocol likely initiated this rapid, compensatory GABA-driven mechanism, which would be expected to reduce the susceptibility of the V1 circuit to the long-term potentiation (LTP) or long-term depression (LTD)-like effects of TBS [37].

Previous studies have primarily focused on chronic, maladaptive visual cortex conditions, such as amblyopia [22,38]. In contrast, our study targeted an acute, non-pathological adaptive response. In conditions like amblyopia, a profound, chronic altered neural state, characterised by disrupted excitation and inhibition balance, already exists [39,40]. This might make the visual cortex inherently more susceptible and responsive to exogenous neuromodulation designed to restore the equilibrium. Our null result is consistent with the possibility that the visual cortex of adults with typical vision, although it retains plasticity mechanisms, was not measurably driven by acute TBS while the system was actively engaged in short-term adaptive compensation for the induced blur. One speculative account, which our behavioural data cannot confirm, is that this reflects the tight regulatory control exerted by inhibitory synapses in V1 to maintain homeostatic set points [36,41], which could make the cortex less readily driven into a new functional state by directional LTP/LTD-like effects [15].

While TBS did not modulate performance, a non-specific improvement in VA over time was observed across stimulation conditions. This improvement, which reflects a recovery of high spatial frequency perception, aligns with the masking hypothesis proposed by Mon-Williams and colleagues [42], who found that 30-minutes of +1.00D blur led to a binocular VA improvement of approximately 0.09 logMAR. In comparison, we observed a smaller improvement of 0.03 logMAR with +1.50D induced blur. This difference in VA improvement is expected because our ‘pre’ baseline was established after an initial 10-minute adaptation period, while Mon-Williams and colleagues [42] measured total gain from a pre-adaptation (0-minute) baseline. As the rate of neural recalibration is steepest at the onset of blur, with significant effects emerging within the first four minutes [26], our result likely captures the incremental neural gain accrued between the 10th and 25th minute of cumulative blur exposure – a state largely preserved during the TBS stimulation interval by an eyes-closed protocol that minimised de-adaptation to clear vision. Mechanistically, the VA improvement was posited to occur via the unmasking of low-contrast, high-frequency components, whereby the visual system reduces the gain of mid-range channels, lowering their “masking” effect on the higher frequency signals critical for acuity.

The absence of any change in contrast sensitivity in our study can be interpreted by considering the influence of adaptation duration on mid-range spatial frequencies. The spatial frequency of the stimulus used in the contrast discrimination task (10 cpd) falls squarely within the mid-range spatial frequency spectrum where Mon-Williams and colleagues [42] observed a reduction in sensitivity – the very mechanism driving the VA improvement – following blur adaptation. Given that baseline contrast threshold measurements were statistically equivalent across conditions, this persistent null result suggests that the 10-minute adaptation period in our study may have been too brief to fully activate or stabilise the mid-range channel gain-reduction mechanism. This short blur adaptation duration may have contributed to the variability observed across our contrast threshold data, as well as the unexpectedly noisy, low-amplitude signals in our sweep VEP data that ultimately rendered the data unanalysable.

Our null finding in contrast sensitivity is incongruent with the initial effect reported by Waterston and Pack [20], who found that cTBS over V1 in typically sighted adults significantly improved accuracy in a coarse orientation discrimination task presented in the peripheral visual field (6° eccentricity) following stimulation. This key difference suggests that the effectiveness of acute V1 neuromodulation may be visuotopically dependent. Our study targeted the V1 representation of central vision using visual functions dependent on high resolution central vision. The high cortical magnification and functional stability of the central visual field relative to the periphery [43–47] may make central vision less susceptible to generalised TBS modulation compared to peripheral vision.

Waterston and Pack [20] also reported that cTBS did not modulate performance on a fine orientation discrimination task tested across multiple eccentric visual field locations. This aligns with our results for high resolution central vision measures, contrast discrimination threshold and VA; suggests that the cTBS effect is task specific. Therefore, the lack of observable results in our tasks and those in the study of Waterston and Pack [20] suggests that TBS may be more suited for modulating low-level mechanisms (like coarse feature detection) rather than complex neural computations required for fine discrimination.

Our null result also raises the question of whether our TBS protocol successfully modulated V1 cortical excitability as intended. Studies on V1 excitability, such as those using the PT, confirm that cTBS can induce a physiological inhibitory effect [17]. However, the relationship between this physiological change and behavioural outcomes is complex. For example, Waterston and Pack [20] found that cTBS improved coarse discrimination. Taken together, these two results suggest a dissociation between the expected inhibitory physiological effect and the facilitatory behavioural change. Unfortunately, we were unable to analyse the VEP data collected due to noise, and as such we were not able to assess whether TBS would produce the expected effect on cortical excitability.

Future research might consider adopting a longer adaptation duration (e.g., ≥ 30 minutes) prior to measuring visual function since that may be necessary to reliably shift the visual system’s state and drive gain-adjustment mechanisms, as suggested by Mon-Williams and colleagues [42]. However, protocols must be designed to capture the window during which the cortex is plastic but still imbalanced, as the visual system compensates and recovers quickly [36,48]. Other promising avenues include combining TBS with PL, as PL actively engages the visual system, potentially increasing its susceptibility to TBS and allowing stimulation to enhance learning-related gains.

### Limitations

This study has several limitations. First, it was not powered a priori to detect small acute effects of TBS. With only twelve participants and substantial interindividual variability in TMS responsiveness, phosphene thresholds, and blur adaptation, the data cannot strongly support the absence of an effect. Consequently, these findings should be interpreted as the absence of a detectable acute effect under these conditions, rather than as evidence that the visual cortex cannot be modulated. Second, we did not obtain a physiological index of stimulation efficacy: phosphene thresholds were recorded only at baseline for dosing, no post-stimulation phosphene threshold was collected, and the sweep VEP data were unusable. We therefore cannot confirm that the TBS protocols modulated V1 excitability, and the behavioural null result must be interpreted with this caveat.

## Conclusion

Visual cortex cTBS and iTBS did not alter short-term blur adaptation measured using contrast discrimination thresholds or VA. The visual cortex of adults with typical vision, while possessing plasticity mechanisms, did not show a detectable response to acute neuromodulation in our behavioural measures. Our findings are best interpreted within the framework of homeostatic plasticity – when the visual system is confronted with a transient sensory challenge, robust, GABA-mediated homeostatic mechanisms rapidly engage to maintain the current functional set point, which may render the circuit less susceptible to the external LTD/LTP-like effects intended by TBS. The effectiveness of TBS is thus likely confined to specific conditions that involve chronically destabilised cortical states (e.g., amblyopia) or highly visuotopically and task-specific context (e.g., coarse discrimination in peripheral vision). We conclude that for adults with typical vision, overcoming this intrinsic stability and leveraging plasticity for functional gain requires future neuromodulation protocols to be actively integrated with task engagement to create the necessary, persistent state of functional imbalance.
