# Supplementary material for: Theta burst stimulation of the visual cortex does not alter contrast sensitivity under optically induced blur
Source: PLoS One. 2026 Sep 1;21(9):e0341942. doi: 10.1371/journal.pone.0341942 (PMC13533367; doi:10.1371/journal.pone.0341942)
Supplement: Supplementary file 1 [file pone.0341942.s002.docx]

# **Response to Reviewers**

Manuscript ID: PONE-S-26-01805

Title: Theta burst stimulation of the visual cortex does not alter contrast sensitivity under optically induced blur

Journal: PLOS ONE

Dear Dr. Che,

We thank you and both reviewers for the constructive evaluation of our manuscript. We have addressed each point raised by reviewers in the point-by-point response below, with page and line numbers corresponding to the revised manuscript with tracked changes.

## **Reviewer #1**

*The final analyzed sample consisted of only 12 participants. This is a very small sample for detecting subtle neuromodulatory effects, particularly given high interindividual variability in TMS responsiveness, known variability in phosphene thresholds, variability in visual adaptation, and modest expected behavioral effects. Underpowered null studies cannot strongly support claims regarding the absence of effect. This is one of the most important limitations and must be explicitly acknowledged.*

We appreciate the reviewer’s insightful comments regarding the challenges of interpreting null results within a small sample with interindividual variability. We acknowledge that the study was not powered a priori to detect small acute effects, and we updated the manuscript to state this explicitly. We have added a dedicated limitation acknowledging that our results should be viewed as an absence of detectable effect under these specific conditions, rather than evidence that no effect exists. To aid in the interpretation of these null results, we now report a sensitivity analysis and provide a Bayesian analysis to quantify the level of evidence supporting the null hypothesis.

**Page 22 (Discussion), lines 444–453:** “First, it was not powered a priori to detect small acute effects of TBS; with twelve participants and the substantial interindividual variability in TMS responsiveness, phosphene thresholds, and blur adaptation, the data cannot strongly support the absence of an effect, and the present finding should be read as the absence of a detectable acute effect under these conditions rather than as evidence that the visual cortex cannot be modulated. Second, we did not obtain a physiological index of stimulation efficacy: phosphene thresholds were recorded only at baseline for dosing, no post-stimulation phosphene threshold was collected, and the sweep VEP data were unusable. We therefore cannot confirm that the TBS protocols modulated V1 excitability, and the behavioural null result must be interpreted with this caveat.”
**Pages 15–16 (Methods), lines 297-301:** “To quantify evidence for the null hypothesis, the active versus sham change was additionally evaluated with a Bayesian paired analysis reporting the Bayes factor in favour of the null (BF_01_). A sensitivity analysis indicated that, with twelve participants at α = 0.05 and 80% power, only a large within-subject effect (dz of approximately 0.89) could be detected.”
**Page 16 (Results), lines 318–320:** “The pre-to-post change did not differ between the active and sham conditions; the Bayesian analysis provided moderate evidence against a differential effect (BF01 = 3.41 for cTBS vs. sham and 3.45 for iTBS vs. sham).”
**Page 17 (Results), lines 339–343:** “The improvement in VA did not differ between the active and sham conditions; the Bayesian analysis provided moderate evidence against a differential effect (BF_01_ = 3.30 for cTBS vs. sham and 3.08 for iTBS vs. sham).”

*The manuscript reports F and p values but lacks effect sizes (partial eta squared for all analyses), confidence intervals, assumptions testing, normality assessment, sphericity assessment, and correction procedures if assumptions were violated.*

In response to the reviewer’s feedback, we have expanded our statistical reporting to improve the clarity and precision of our results. Specifically, we now report partial eta squared for all effects and 95% confidence intervals for all condition means and pre-post change scores. For statistical assumptions, Mauchly’s test indicated that sphericity was not violated for both outcome measures (contrast, W = 0.58, p = 0.064; visual acuity, W = 0.90, p = 0.588); Greenhouse-Geisser corrected p values are reported and do not change any conclusion. Shapiro-Wilk tests on the change scores were non-significant across all visual acuity and active contrast conditions. Although the sham contrast change deviated from normality (W = 0.80, p = 0.009) owing to two outlying values, a supplementary Wilcoxon signed-rank check returned the same null conclusion, supporting the consistency of the primary analysis.

**Page 15 (Methods), lines 294–297:** “Partial eta squared (ηp²) is reported for all effects. Normality of the change scores was assessed with the Shapiro-Wilk test and sphericity with Mauchly’s test; Greenhouse-Geisser corrected degrees of freedom are reported where sphericity was violated.”
**Page 16 (Results), lines 309–317:** “… a statistically non-significant interaction of Stimulation × Time (F(2, 22) = 0.03, p = 0.96, partial ηp² = 0.003) … No main effects of Stimulation (F(2, 22) = 1.07, p = 0.35, partial ηp² = 0.089) or Time (F(1, 11) = 0.07, p = 0.79, partial ηp² = 0.007) were observed. The mean ± SEM contrast thresholds were: cTBS (Pre: 0.22 ± 0.03, 95% CI 0.15–0.29 vs. Post: 0.23 ± 0.03, 95% CI 0.15–0.31), iTBS (Pre: 0.20 ± 0.01, 95% CI 0.16–0.24 vs. Post: 0.20 ± 0.03, 95% CI 0.14–0.27), and sham (Pre: 0.21 ± 0.03, 95% CI 0.15–0.29 vs. Post: 0.21 ± 0.02, 95% CI 0.16–0.27).”
**Pages 16–17 (Results), lines 320–323:** “Sphericity was not violated (Mauchly, p = 0.064) and Greenhouse-Geisser correction did not alter this conclusion. Change scores were normally distributed for the active conditions; the sham change deviated from normality (Shapiro-Wilk, p = 0.009), a Wilcoxon signed-rank test confirmed the null result.”
**Page 17 (Results), lines 330–343:** “… no significant interaction of Stimulation × Time (F(2, 22) = 0.12, p = 0.88, partial ηp² = 0.011) … a significant main effect of Time (F(1,11) = 6.30, *p* = 0.029, partial ηp² = 0.364) … Sphericity was not violated (Mauchly, p = 0.59), and all change scores were normally distributed (Shapiro-Wilk, all *p* > 0.15).”

*The manuscript repeatedly concludes that the visual cortex is “resistant” to acute neuromodulation, blur adaptation represents a “maximum response,” and TBS is ineffective. However, a null result in a small behavioral study does not demonstrate absence of physiological modulation. The conclusions must be rewritten more cautiously.*

We have rewritten the Conclusion so that it reports only that no behavioural change was observed following acute TBS, and we no longer describe the visual cortex as "resistant" to neuromodulation, blur adaptation as a "maximum response", or TBS as ineffective. The revised Conclusion makes explicit that a null behavioural result in a small sample does not demonstrate an absence of physiological modulation.

**Page 19 (Discussion), lines 377–383:** “Our null result is consistent with the possibility that the visual cortex of adults with typical vision, although it retains plasticity mechanisms, was not measurably driven by acute TBS while the system was actively engaged in short-term adaptive compensation for the induced blur. One speculative account, which our behavioural data cannot confirm, is that this reflects the tight regulatory control exerted by inhibitory synapses in V1 to maintain homeostatic set points, which could make the cortex less readily driven into a new functional state by directional LTP/LTD-like effects.”
**Page 23 (Conclusion), lines 457–462:** “The visual cortex of adults with typical vision, while possessing plasticity mechanisms, did not show a detectable response to acute neuromodulation in our behavioural measures. Our findings are best interpreted within the framework of homeostatic plasticity — when the visual system is confronted with a transient sensory challenge, robust, GABA-mediated homeostatic mechanisms rapidly engage to maintain the current functional set point, which may render the circuit less susceptible to the external LTD/LTP-like effects intended by TBS.”

*The study fundamentally assumes that cTBS and iTBS successfully modulated cortical excitability. However, no pre/post phosphene threshold analysis was reported, and no physiological verification of stimulation efficacy exists. The discussion should explicitly recognize that the central experimental manipulation lacks physiological confirmation.*

We agree that the lack of direct physiological validation is a critical point for interpreting our findings. As noted, phosphene thresholds were measured only at baseline for dosing purposes, and no post-stimulation thresholds were obtained. Furthermore, while sweep VEP data were collected, they had to be excluded for excessive noise, leaving us without a direct physiological confirmation of V1 modulation. We have now explicitly acknowledged this as a key limitation and have integrated this caveat into our discussion to ensure a more cautious interpretation of the behavioural results.

**Page 22 (Discussion), lines 449–453:** “Second, we did not obtain a physiological index of stimulation efficacy: phosphene thresholds were recorded only at baseline for dosing, no post-stimulation phosphene threshold was collected, and the sweep VEP data were unusable. We therefore cannot confirm that the TBS protocols modulated V1 excitability, and the behavioural null result must be interpreted with this caveat.”

## **Reviewer #2**

We thank Reviewer 2 for judging the design reasonable and the null result potentially suitable for PLOS ONE, and for a detailed set of recommendations that we address point by point.

*The final sample of 12 participants is small, no power calculation or smallest detectable effect is reported, and yet the negative result is presented with more confidence than the data allow. The authors should add effect sizes, 95% CIs, and ideally an equivalence or Bayesian analysis.*

In response to the suggestion to enhance our statistical rigour, we have implemented all of the requested metrics. Specifically, partial eta squared is now reported for every effect to provide a clear measure of effect size. To directly address the null results, we have also supplemented our approach with a Bayesian paired analysis of the active-versus-sham change. This analysis yielded Bayse Factors that provide moderate evidence for the absence of a differential effect across both contrast thresholds and visual acuity outcomes.

**Page 15 (Methods), lines 294-301:** “Partial eta squared (ηp²) is reported for all effects. Normality of the change scores was assessed with the Shapiro-Wilk test and sphericity with Mauchly’s test; Greenhouse-Geisser corrected degrees of freedom are reported where sphericity was violated. To quantify evidence for the null hypothesis, the active versus sham change was additionally evaluated with a Bayesian paired analysis reporting the Bayes factor in favour of the null (BF_01_). A sensitivity analysis indicated that, with twelve participants at α = 0.05 and 80% power, only a large within-subject effect (d_z_ of approximately 0.89) could be detected.”
**Page 16 (Results), lines 318–320:** “The pre-to-post change did not differ between the active and sham conditions; the Bayesian analysis provided moderate evidence against a differential effect (BF01 = 3.41 for cTBS vs. sham and 3.45 for iTBS vs. sham).”
**Page 17 (Results), lines 340–342:** “The improvement in VA did not differ between the active and sham conditions; the Bayesian analysis provided moderate evidence against a differential effect (BF01 = 3.30 for cTBS vs. sham and 3.08 for iTBS vs. sham).”

*Assumptions, individual-level change data, psychophysical fit quality, parameter constraints, lapse rates, and any excluded sessions should all be documented.*

We thank the reviewer for identifying these areas for further documentation. We have now detailed each item in the revised manuscript: (a) Assumptions: we have added formal reporting of Shapiro-Wilk and Mauchly’s tests to confirm that assumptions for our parametric analyses were met; (b) Individual change data: to improve transparency regarding inter-individual variability, Figures 2 and 3 now include connected individual data points alongside group means; (c) Psychophysical fit quality: we now report that the Weibull psychometric functions provided a high quality of fit; (d) Parameter constraints: we have clarified that the guess rate was fixed at 0.5 and the lapse rate at 0.02, allowing for more stable estimates of the free parameters, threshold and slope; (e) Excluded sessions: we have, in our previous manuscript, documented that only one participant was excluded following a technical error involving an incorrect blinding code (lines 185-187).

**Page 15 (Methods), lines 281–288:** “A four-parameter Weibull psychometric function was fitted using weighted nonlinear least squares (SciPy curve_fit), with threshold (κ) and slope (α) as free parameters; the guess rate (γ) was fixed at 0.5, corresponding to chance performance in the two-alternative forced-choice task, and the lapse rate (λ) was fixed at 0.02. Contrast threshold was defined as the stimulus level corresponding to 76% correct performance. This threshold, representing the inverse of contrast sensitivity [33], was the key psychophysical outcome measure used to evaluate any acute changes in visual performance following TBS. Weibull functions fitted the data well (median R² = 0.88). ”
**Page 16 (Methods), lines 295–297:** “Normality of the change scores was assessed with the Shapiro-Wilk test and sphericity with Mauchly’s test; Greenhouse-Geisser corrected degrees of freedom are reported where sphericity was violated.”

*The mechanistic discussion invoking homeostatic plasticity, GABA-mediated inhibition, and resistance to neuromodulation goes well beyond what was actually measured, since the VEP data were excluded and no post-stimulation physiological marker was obtained; these should be reframed as speculative hypotheses.*

We agree that without physiological confirmation, our mechanistic discussion should be framed as a hypothesis. We have now reframed the homeostatic plasticity, GABA, and LTP/LTD account as a speculative hypothesis and ensured this more cautious tone is maintained throughout the Discussion and Conclusion.

**Page 19 (Discussion), lines 377–383:** “Our null result is consistent with the possibility that the visual cortex of adults with typical vision, although it retains plasticity mechanisms, was not measurably driven by acute TBS while the system was actively engaged in short-term adaptive compensation for the induced blur. One speculative account, which our behavioural data cannot confirm, is that this reflects the tight regulatory control exerted by inhibitory synapses in V1 to maintain homeostatic set points, which could make the cortex less readily driven into a new functional state by directional LTP/LTD-like effects.”

*The authors should clarify what changed from the preregistered protocol, whether VA was a prespecified outcome or adopted post-hoc, and whether the analysis plan was modified after inspecting the data.*

We have updated the Methods section to more explicitly label our primary and post-hoc outcomes. We now state clearly that while the contrast threshold was the pre-registered primary outcome, the VA analysis was a post-hoc addition. We also explicitly confirm that the analysis for the primary outcome was not altered after data inspection. The exclusion of the sweet VEP data is also detailed alongside these points to ensure all deviations are transparently disclosed in one location.

**Page 6 (Methods), lines 116–124:** “Deviations from the pre-registered protocol included the exclusion of sweep VEP data; despite adherence to ISCEV standards using full-field vertical square wave gratings, the induced blur and 60% contrast conditions resulted in excessive noise and low VEP amplitudes, making the data unusable for analysis. Furthermore, while the contrast discrimination threshold was the primary pre-registered outcome, the VA analysis was adopted post-hoc. VA was originally collected as a blur adaptation check, and the final stabilised value (the last time point of the adaptation period, at 10 or 12 minutes) was subsequently used as a dependent variable in the analysis. We confirm that the analysis plan for the primary contrast threshold outcome was not modified after inspecting the data.”

*Individual pre- and post-stimulation VA and contrast threshold values must be made available, as the current Data Availability Statement is inconsistent with a manuscript that reports only summary statistics.*

We will deposit the individual pre and post visual acuity and contrast threshold values in the study’s OSF project to ensure consistency with the Data Availability Statement upon manuscript acceptance.

*The blinding check after each session should be reported, the 24-hour washout justified in relation to expected TBS effect duration, and order or carryover effects addressed.*

We agree that blinding and carryover are critical considerations for this design. While a formal post-session blinding check was not collected, we acknowledge this as a limitation. To ensure the integrity of the double-blind, we employed physically indistinguishable active and placebo coil faces (Cool-B70 A/P) and used a blinding code held by a team member naïve to the study aims. To empirically address carryover effects, we compared baseline measures across all conditions. The absence of significant differences in baseline contrast thresholds (F(2, 22) = 0.36, p = 0.69, partial eta squared = 0.032) and visual acuity (F(2, 22) = 2.49, p = 0.10, partial eta squared = 0.185) confirms that there were no measurable cumulative effects between sessions.

**Page 12 (Methods), lines 218 and 227–228:** “… using a Cool-B70 A/P coil, which incorporates both an active and a placebo side that are physically indistinguishable to the experimenter, thereby ensuring experimenter blinding.” … “A double-blinded design was maintained using a pre-generated randomization list, with associated blinding codes, managed by a team member naïve to the experimental aims.”
**Page 16 (Results), lines 303–306:** “RM ANOVA on pre-stimulation contrast thresholds showed no statistically significant baseline differences (F(2,22) = 0.36, p = 0.69, partial ηp² = 0.032) … RM ANOVA on pre-stimulation VA revealed no statistically significant baseline differences (F(2,22) = 2.49, p = 0.10, partial ηp² = 0.185).”

*Several language errors also need correcting (“supports for the notion,” “specific visual tasks trainings,” “Despite these inconsistency results,” “led to led to”).*

All four have been corrected: “strongly supports for the notion” to “strongly supports the notion”; “specific visual tasks trainings” to “training on specific visual tasks”; “Despite these inconsistency results” to “Despite these inconsistent results”; and “led to led to” to “led to”. We also corrected “potentials of NIBS” to “potential of NIBS” and proofread the complete manuscript for similar errors.

Yours sincerely,

Crystal Suei Cheng Wong

(on behalf of the authorship team)

School of Optometry, The Hong Kong Polytechnic University
